# Spinal Tuberculosis (Pott’s Disease): A Case Report and Clinical Considerations

**DOI:** 10.7759/cureus.73903

**Published:** 2024-11-18

**Authors:** Win Lae Lae Aung, Noim Jibon, Lama Rawashdeh, Ali Raheem, Marjorie Jaffet, Shahid Nasim

**Affiliations:** 1 Medicine, Diana, Princess of Wales Hospital, Grimsby, GBR; 2 Medicine, Southend University Hospital, Mid and South Essex NHS Foundation Trust, Southend-on-Sea, GBR; 3 Internal Medicine, Basildon University Hospital, Mid and South Essex NHS Foundation Trust, Basildon, GBR; 4 Gastroenterology, Southend University Hospital, Mid and South Essex NHS Foundation Trust, Southend-on-Sea, GBR; 5 General Medicine, Southend University Hospital, Mid and South Essex NHS Foundation Trust, Southend-on-Sea, GBR; 6 Internal Medicine, Southend University Hospital, Mid and South Essex NHS Foundation Trust, Southend-on-Sea, GBR

**Keywords:** anti-tuberculosis treatment, extra-pulmonary tb, pott’s spine, psoas abcess, spinal tuberculosis:, tb – tuberculosis

## Abstract

Pott's disease (PD), also known as spinal tuberculosis, accounts for an extremely low percentage of all tuberculosis (TB) cases and typically manifests secondary to an extra-spinal infection through the hematogenous spread. The thoracolumbar vertebrae are the most affected sites in PD, although other spinal regions can also be involved, albeit less frequently. Back pain is the predominant presenting symptom. Systemic symptoms such as fever and weight loss may occur in PD but are more commonly observed in patients with disseminated disease or concurrent extra-spinal TB. This report presents the case of a 26-year-old Asian woman, born outside the United Kingdom, with no significant past medical history or recent travel to TB-endemic areas, who was diagnosed with spinal TB following a series of medical evaluations. MRI of the spine revealed findings highly suggestive of tuberculous spondylitis. She is currently undergoing treatment and showing significant improvement. This case underscores an atypical patient profile for spinal TB, highlighting the necessity for awareness and consideration of this diagnosis even in unusual patient demographics.

## Introduction

The United Kingdom (UK) is considered a low-incidence tuberculosis (TB) country, with rates of fewer than 10 cases per 100,000 people [1]. The first modern case of spinal TB, also known as Pott's disease, was described in 1779 by Percival Pott [2]. Despite the overall incidence of spinal TB remaining stable or declining in most European countries, there is an increasing proportion of cases among foreign-born populations. In the UK, TB is rare, with a notification rate of 8.4 cases per 100,000 population [3]. 

While the precise global incidence of spinal TB is not well-documented, certain regions have reported significant increases, particularly due to factors such as immigration and the HIV epidemic [1]. According to the World Health Organization (WHO), in 2023, an estimated 10.8 million people worldwide developed TB, with approximately 1.25 million TB-related deaths (including 161,000 people with HIV) [4]. This epidemiological shift underscores the importance of heightened vigilance and targeted public health strategies to address TB in both low- and high-incidence regions [5].

In this report, we present the case of a young Asian woman who, although born outside the UK, had no medical history or recent travel to TB-endemic areas.

## Case presentation

A 26-year-old woman presented with worsening lower back pain and a general feeling of unwellness. She had a history of chronic lower back pain for the past year with radiation down left leg, which had shown partial improvement with physiotherapy. Additionally, she reported an unintentional weight loss of around 10 kg over the same period. Blood tests indicated iron deficiency anaemia, though inflammatory markers were normal. 

**Figure 1:**
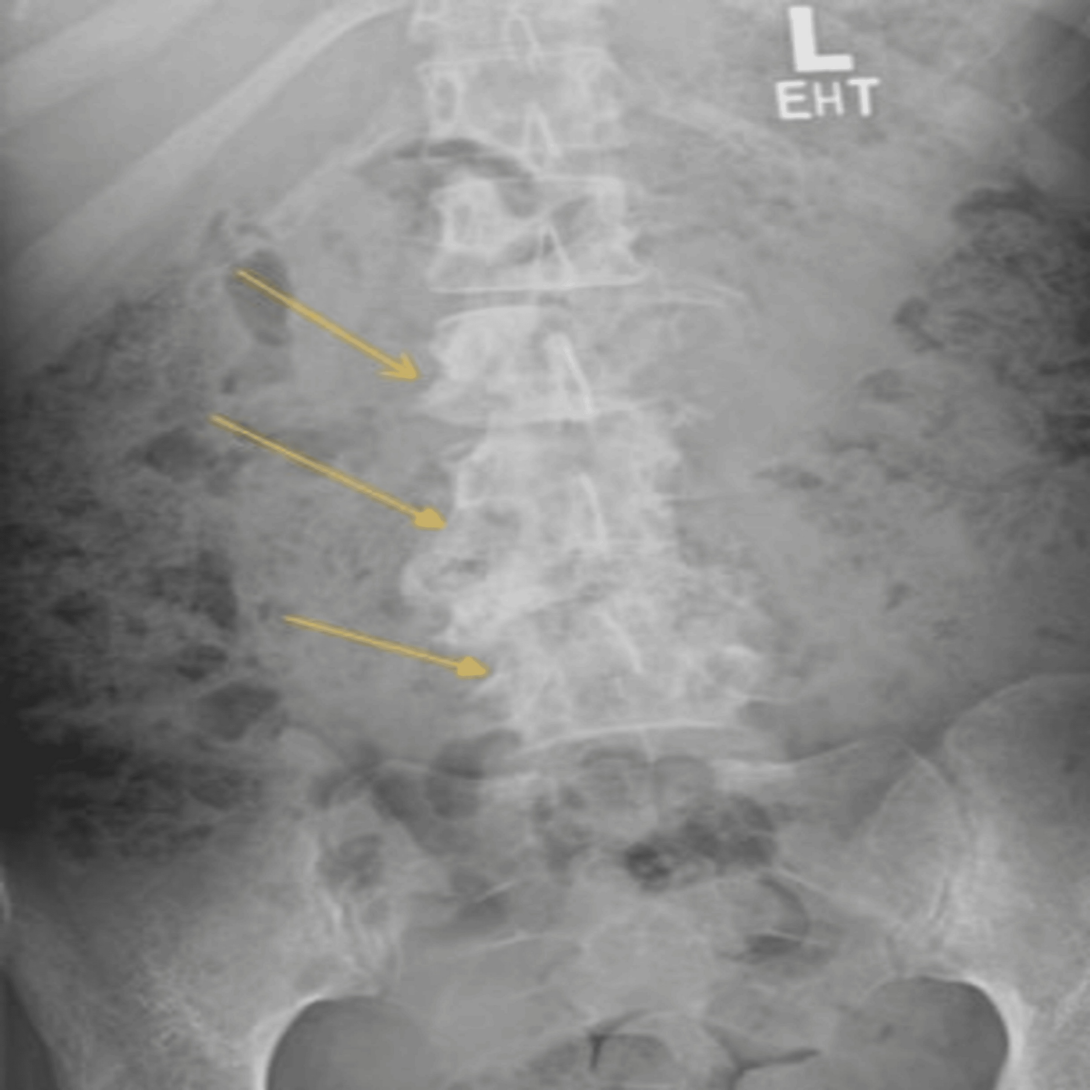
X-ray spine showing complete destruction of the left L2 and L4 vertebral bodies and a severe compression fracture at L4

**Figure 2:**
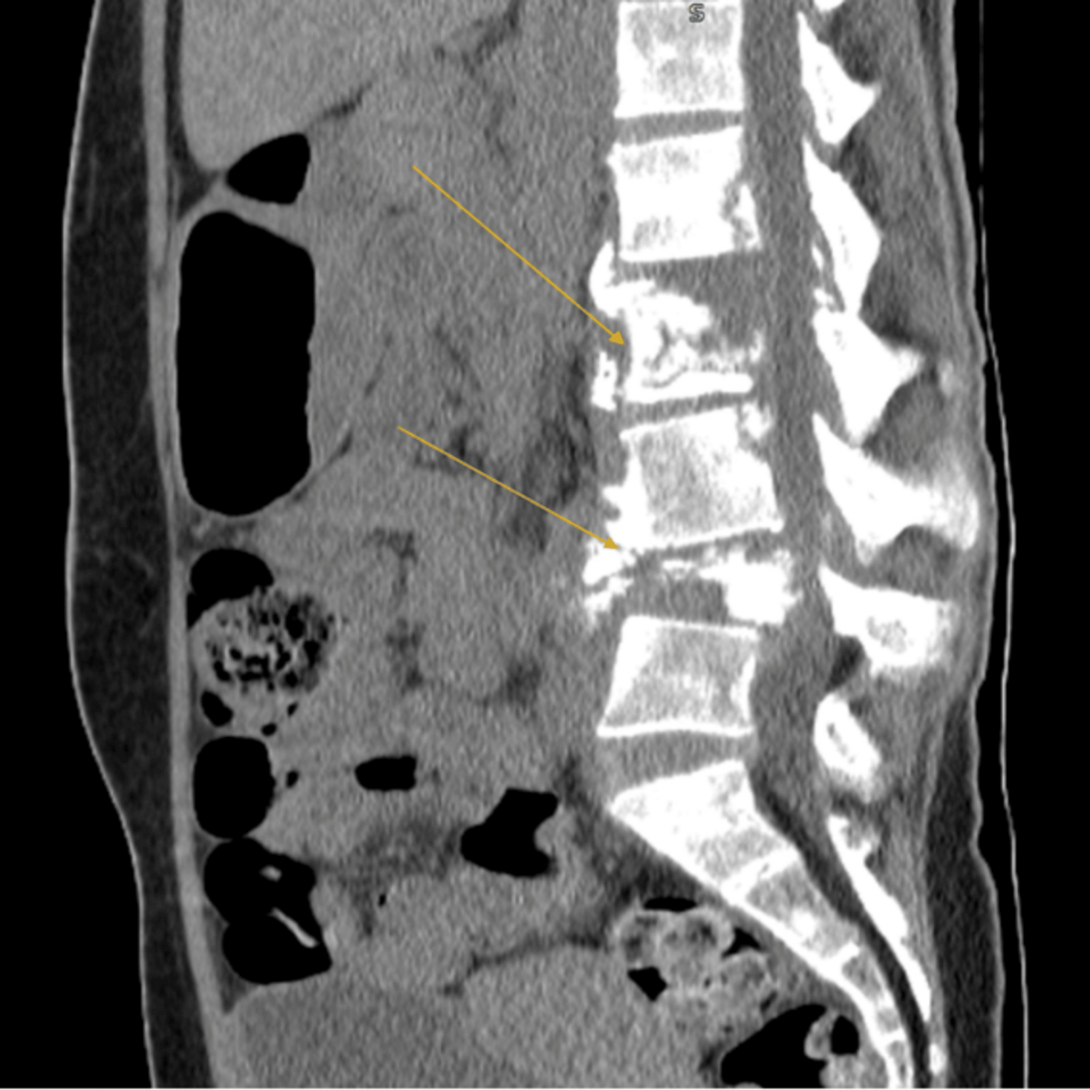
MRI scans of the cervical, thoracic, lumbar, and sacral spine highly suggestive of tuberculous spondylitis, with multilevel spinal canal compromise from T11 to L5

**Figure 3:**
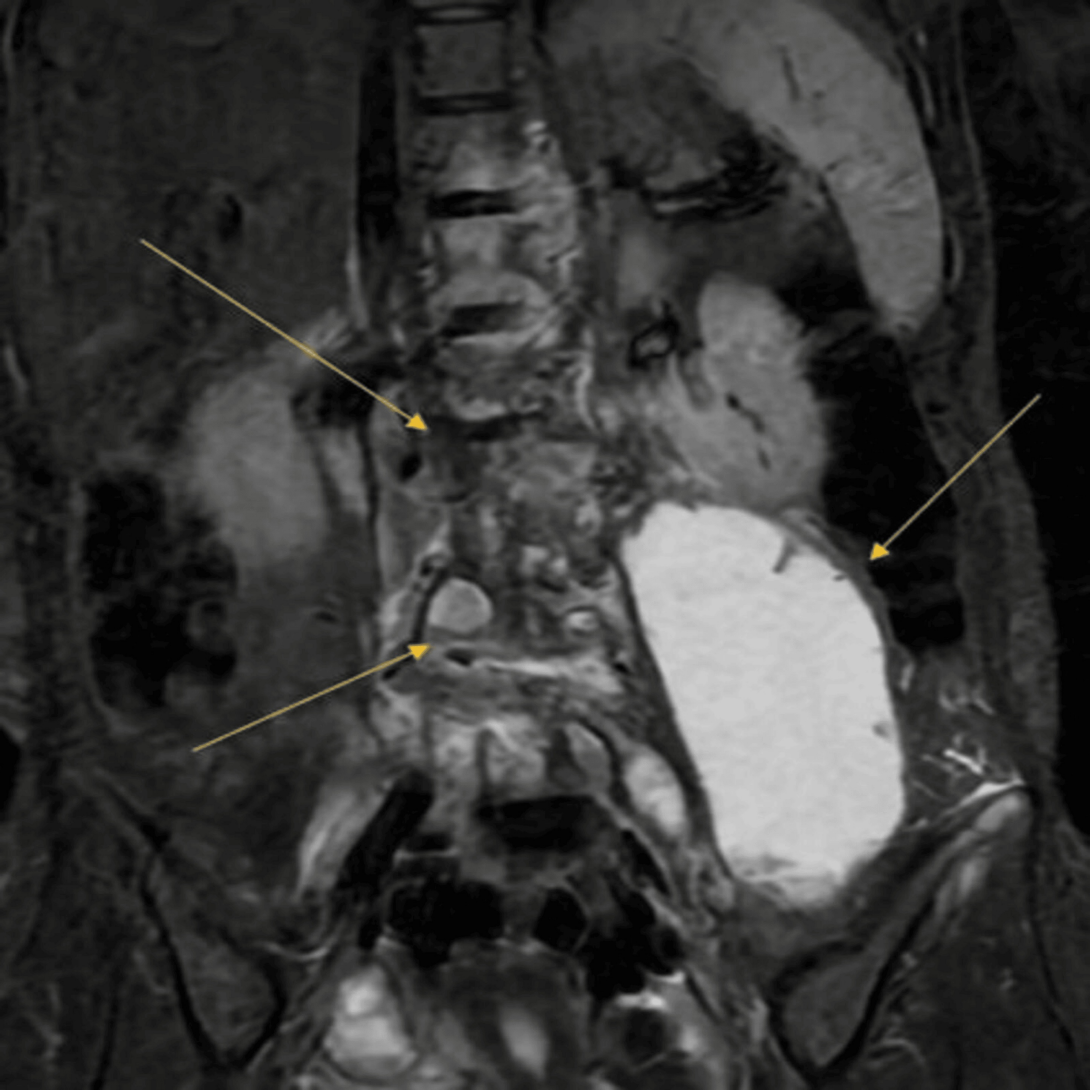
A large psoas abscess extending to the iliacus muscle

An X-ray of the lumbar spine showed complete destruction of the left L2 and L4 vertebral bodies and a severe compression fracture at L4 (Figure 1) . MRI scans of the cervical, thoracic, lumbar, and sacral spine revealed findings highly suggestive of tuberculous spondylitis, with multilevel spinal canal compromise from T11 to L5 (Figure 2). The most severe impact was at L4, resulting in severe central canal stenosis due to complete vertebral collapse. A large psoas abscess extending to the iliacus muscle was also identified (Figure 3). Cultures were taken from the abscess which approved positive for acid fast bacili, tuberculose by whole genome sequencing (WGS).

The patient began anti-tuberculosis treatment with HRZE (isoniazid, rifampicin, pyrazinamide, and ethambutol). Pre-ethambutol eye check was done. The large left psoas abscess was drained under ultrasound guidance. 

Eighteen days after the abscess was drained, she presented again with worsening back pain. A subsequent CT of the lumbar spine revealed larger bilateral psoas abscesses (left greater than right) and an epidural abscess. The left psoas abscess was drained again. The Neurosurgery team was also included in her care to ensure the neurosurgical requirement was not required for her back pain and spinal stenosis. The patient continued to follow up with an Infectious Disease specialist and recovered well, with no side effects from the anti-TB treatment. 

A repeat MRI done after eight weeks of HRZE treatment showed an overall interval reduction in the size of extradural subligamentous collections and psoas abscesses. There was interval improvement of the spinal canal stenosis. Her back pain was settled without requiring any analgesia. She was treated with HRZE (isoniazid with pyridoxine, pyrazinamide, rifampicin, and ethambutol)) for two months, followed by continuous treatment with isoniazid (with pyridoxine) and rifampicin for an additional 10 months, as per the Infectious Disease specialist's guidance. She is still undergoing treatment.

## Discussion

Pott’s disease, or spinal TB, is a rare form of extrapulmonary TB that constitutes less than 1% of all TB cases globally [6]. The incidence of spinal TB in the UK as of 2021 was 4.3% [7]. It typically affects the thoracolumbar vertebrae and is often secondary to hematogenous spread from a primary site of infection, usually the lungs. The incidence of spinal TB in low-incidence countries like the UK has been rising, primarily due to factors such as increased immigration from TB-endemic regions and the HIV epidemic, which increases susceptibility to TB infections [1,8]. 

This case highlights several critical aspects of spinal TB, including the diagnostic challenges and complexities of management. This case presentation with chronic lower back pain and significant unintentional weight loss, along with iron deficiency anemia and normal inflammatory markers, could be easily misattributed to more common conditions, leading to potential delays in diagnosis. The presence of chronic symptoms and weight loss in this patient should raise suspicion for a more insidious process such as TB, particularly considering the global epidemiology of the disease [9].

Imaging played a crucial role in the diagnosis. The initial X-ray of the lumbar spine revealed significant pathological changes which were confirmed further by the MRI highlighting the complications of TB. 

The initial management strategy involved starting the patient on a standard anti-tuberculous regimen (HRZE). This regimen is recommended for treating spinal TB and aimed at eradicating the infection and preventing further progression of the disease which can be confirmed by the patient’s recovery progress [10]. 

Further monitoring was essential due to the recurrence of the psoas abscess even after the first drain. Additionally, the large left psoas abscess was drained under ultrasound guidance, which is a critical step in managing such complications to reduce the risk of sepsis and enhance recovery. Despite initial treatment, the patient presented again with worsening back pain, and an interval CT scan revealed further complications. Because of the chronic and persistent nature of TB, and the challenges in eradicating infection, The need for repeat drainage of the left psoas abscess highlights the importance of close monitoring and prompt intervention in the management of spinal TB [10]. 

Multidisciplinary team (MDT) involvement is crucial. The involvement of the neurosurgical team and their decision not to pursue surgical intervention is notable. Surgical management of spinal TB is typically reserved for cases with severe neurological deficits, spinal instability, or failure of medical management [11]. In the current patient’s case, the absence of severe neurological impairment or spinal instability justified a conservative approach focused on medical therapy and abscess drainage. This decision aligns with recommendations from surgeons based on MDT involvement that emphasize the role of surgery primarily in cases with specific indications [12]. 

The ongoing follow-up with an infectious disease specialist is crucial for monitoring treatment response and managing potential side effects of anti-tuberculous therapy [12]. This patient’s recovery without significant side effects from the medication is a positive outcome, reflecting the effectiveness of the current treatment regimen. 

This case underscores the importance of considering spinal TB in the differential diagnosis of chronic back pain, especially in patients with atypical profiles or nonspecific symptoms [13]. It also highlights the need for a multidisciplinary approach involving infectious disease specialists, radiologists, and surgeons to ensure comprehensive care. Early diagnosis, appropriate therapeutic interventions, and careful monitoring are essential to effectively manage this rare but serious condition [13]. 

## Conclusions

In conclusion, raising awareness of atypical presentations of tuberculous spondylitis is essential. Early diagnosis is key to ensuring appropriate treatment and preventing serious complications and long-term morbidity. In many cases, a definitive diagnosis can only be achieved through biopsy and histopathological analysis.

Our patient is responding well to ongoing treatment, which will continue for one year. She has not required surgical intervention, underscoring the significance of timely medical management in halting disease progression.
